# Parental Response Style to Adolescent Self-Harm: Psychological, Social and Functional Impacts

**DOI:** 10.3390/ijerph182413407

**Published:** 2021-12-20

**Authors:** Michelle L. Townsend, Caitlin E. Miller, Emily L. Matthews, Brin F. S. Grenyer

**Affiliations:** 1 School of Psychology, University of Wollongong, Wollongong, NSW 2522, Australia; cm516@uowmail.edu.au (C.E.M.); emilym@uow.edu.au (E.L.M.); grenyer@uow.edu.au (B.F.S.G.); 2 Illawarra Health and Medical Research Institute, University of Wollongong, Wollongong, NSW 2522, Australia

**Keywords:** adolescent, self-harm, NSSI, parenting, mental health, response style

## Abstract

Adolescent self-harm is a significant public health issue. We aimed to understand how parent stress response styles to their child’s self-harm affects their wellbeing and functioning and the wider family. Thirty-seven participants in Australia (parents; 92% female) completed a mixed methods survey regarding their adolescent child’s self-harm. We conducted Pearson zero-order correlations and independent *t*-tests to examine the impact of parent response style on their quality of life, health satisfaction, daily functioning, and mental health. We also used thematic analysis to identify patterns of meaning in the data. Two-thirds of participants reported mental ill health and reduced functional capacity due to their adolescent’s self-harm. Parents with a more adaptive response style to stress had better mental health. Qualitative analyses revealed parents experienced sustained feelings of distress and fear, which resulted in behavioural reactions including hypervigilance and parental mental health symptoms. In the wider family there was a change in dynamics and parents reported both functional and social impacts. There is a need to develop psychological support for the adolescent affected and parents, to support more adaptive response styles, and decrease the negative effects and facilitate the wellbeing of the family unit.

## 1. Introduction

Self-harm in adolescents is a growing international issue, with an average lifetime prevalence of 16–18% for 11–18 year-olds [1,2]. Self-harm is considered intentional self-injury or poisoning [3] and typically begins in early adolescence [1]. Self-harm is a maladaptive behaviour that often serves several intrapersonal and interpersonal functions for adolescents [4]. A meta-synthesis of twenty qualitative studies which focused on the experience of self-harm for young people identified four primary functions of self-harm [5]. This included self-harm as a way to feel relief from intolerable emotions and to experience a feeling of being alive, to control difficult emotions and experiences, to express difficult feelings and protect others from anger, and to identify and connect with others. Self-harm can also occur with suicide intent or ambivalence towards suicide, and the frequency of these intentions may vary, or there may be common motives for both self-harm and suicide [3]. The family environment is important to the development and maintenance of self-harm in adolescence [6,7,8,9]. For example, a longitudinal study of 1973 adolescents aged 12 to 18 years found the family environment to be a salient factor in self-harm [6]. Supportive family environments were associated with cessation of behaviours whereas unsupportive environments contributed to maintenance of the behaviours. Our study aims to further investigate the relationship between adolescent self-harm and the family environment by focusing on the perspective of parents.

When understanding adolescent self-harm within a family context, it is important to recognise the interactional nature of the relationship. Stress-diathesis models acknowledge the interaction of environmental and individual factors, in that stressful environmental factors such as bullying and dysfunctional family interactions may increase the likelihood of vulnerable individuals engaging in self-harm to tolerate overwhelming emotions [10,11,12]. Further, family systems theory highlights the interdependent nature of the family [13,14]. Family relationships, including those between children and parents, are reciprocal and the tensions and anxieties of one family member are experienced in some way by other family members [13]. The interdependent relationships between parents and adolescents regarding self-harm means a distress response from one family member impacts other family members and their own responses. A review by Arbuthnott and Lewis [15] identified several risk factors for self-harm within the family setting and the parent-child relationship. This included socio-economic status, parental mental health, relationship quality between parent and child, forms of discipline and control within the family, and adverse childhood experiences. In addition, research suggests parental mental ill-health can negatively impact on children in multiple ways, including increasing the likelihood of mental health symptoms and disorders, which may be not just due to a genetic component but also the disruptions to, and styles of, parenting [16]. Specifically in borderline personality disorder (BPD), the mental health of parents has been found to be a predictor of mental ill-health in children [17,18]. Parenting style may also influence the commencement and continuity of self-harm. An authoritarian parenting style, characterised by low emotional support and high control of adolescent behaviour, was found to be a risk factor for self-harm [8]. Furthermore for adolescents, a perceived lack of emotional support by their parents had a direct effect on the frequency of self-harm, and perceived parental criticism had an indirect effect on self-harm frequency [7]. This suggests that perceived invalidation and critical family environments may contribute to adolescent self-harm.

While parental behaviours can play a role in the development of self-harm behaviours, parents are also significantly impacted by adolescent self-harm [15,19]. Parents face challenges in supporting their child who self-harms, experiencing increased negative emotions, an absence of social and professional support and subsequent increased parenting burden and stress [20]. The impact of adolescent self-harm on parents can result in decreased psychological wellbeing and functioning, uncertainty regarding effective parenting styles, and limited resources to support both parents and adolescents [21,22,23]. Mothers of children who have engaged in self-harm reported feelings of guilt, shame, and embarrassment, became hypervigilant to self-harm, and reduced focus on both work and other family members [24]. In a similar study, parents reported intense emotions surrounding their child’s self-harm behaviours and expressed a need for more information regarding self-harm in general and management of self-harm within the home setting [25].

Research suggests that parent-child relationships can change following the immediate response to discovery of their child’s self-harm. Kelada et al. [21] identified initial feelings of shock, fear, grief, and anger for parents, followed by attempts to communicate calmly to the young person and identify reasons for self-harm. The authors also noted various changes in the parent–child relationship and difficulties interacting appropriately with their child following self-harm. Byrne et al. [25] further found that despite parents identifying the importance of open communication between them and their child, parents found it difficult to parent effectively following self-harm behaviours. The stress response style of parents in response to a child who engages in self-harm has also been investigated. According to the model of stress response, there are both voluntary engagement and disengagement responses and involuntary responses to stressors. Voluntary engagement responses to stress include conscious regulation of cognitive, behavioural, emotional, and physiological responses using both primary coping strategies such as problem-solving and secondary coping strategies such as acceptance or distraction. Disengagement responses include factors such as denial and withdrawal [26,27]. Often these response styles are categorised into adaptive and maladaptive coping strategies where approaches such as avoidance, externalising, and withdrawal are often considered maladaptive, and strategies such as problem-solving and emotional regulation are considered adaptive [27,28]. Whitlock et al. [22] analysed differences in secondary stress (also termed caregiver strain) between parents of adolescents who did and did not engage in self-harm. They reported that having a child who self-harms resulted in added stress for parents, particularly subjective internal stress including feelings of blame, guilt, and regret. It is likely that the way parents are personally impacted by their child’s self-harm affects their capacity to parent effectively. In summary, research indicates the complexity of interpersonal stressors and the relationships between these factors in relation to adolescent self-harm [29].

The studies to date have been vital in building our understanding the impact of adolescent self-harm on parents. However, this literature can be expanded in several areas. Firstly, the available research has begun to consider how parental response style may influence adolescent self-harm behaviours [7,8,30], but this can be extended to consider how parental stress response style may influence the impact of self-harm on parents. Research indicates that involuntary response styles predict poorer health and wellbeing, including internalising problems such as symptoms of depression and anxiety [31]. Further, involuntary maladaptive coping to a significant stressor has been linked to poorer functioning [32]. Given the understanding that adolescent self-harm is a significant stressor to parents, the study by Whitlock et al. [22] can be expanded upon by attempting to understand the relationship between a parent’s response to secondary stress (caregiver strain) and their ability to function effectively to support their adolescent. Specifically, it is possible that parents with less adaptive response styles to stress will be impacted more severely in terms of their own psychological and functional health by adolescent self-harm. Secondly, Morgan et al. [33] and Ferrey et al. [23] reported that parents of adolescents who self-harm frequently endorsed low levels of social support. One recent study has also investigated the experiences and needs of parents and carers of young people who self-harm [34]. It was found that parents express the need for greater support to help them support their child, as well as access to resources that increase their knowledge and understanding of self-harm. It is important to understand further how adolescent self-harm and these low levels of social support impact the functioning of parents in better addressing this difficulty.

Therefore, in the current study, we aim to further investigate the dynamic relationship between adolescent self-harm and parents. Family systems theory identifies the interdependent nature of the family unit and specifically the reciprocal relationship between distress and response to distress, and we aim to further understand these patterns in relation to adolescent self-harm from the perspective of parents. Specifically, we aim to understand: (1) parent perceptions of adolescent self-harm and how this affects their own wellbeing and functioning by investigating parents’ quality of life, health satisfaction, capacity to undertake everyday roles, and mental health; (2) how parents conceptualise their own responses to adolescent self-harm and relationship between stress response style and parent’s distress and dysfunction; (3) how adolescent self-harm impacts on parents, siblings, the wider family, and community engagement, as perceived by parents.

We will investigate these aims by extending upon studies undertaken to date by using a concurrent mixed-methods triangulation approach to capture both quantitative and qualitative data weighted equally in the analysis and interpretation process [35,36]. This approach employed a questionnaire administered to participants in Australia with measures which captured quantitative responses and questions requiring qualitative responses. In order to investigate aims 1 and 2, we will use quantitative data from parents to explore the impact on wellbeing, and elucidate the role of response style and possible relationship with the impact of adolescent self-harm on parents. We hypothesise that as a group, parents of adolescents who self-harm will experience symptoms of anxiety and depression, high levels of stress, poor quality of life and health satisfaction, and significant impacts to personal life and functioning. We further hypothesise that parents with less adaptive stress response styles will experience greater dysfunction and poorer mental health than those that are able to respond to stress in a more adaptive manner. The third aim will utilise qualitative data by analysing key themes present in responses from parents whose adolescent engaged in self-harm to understand the impact of self-harm more fully on the parent, their wider family, and their social and support needs.

## 2. Materials and Methods

### 2.1. Participants

Convenience sampling was used to identify a sample of parents with children currently aged between 12 to 18 years who had engaged in self-harm behaviours (there was no imposed limit on whether self-harm was historical or current). To collect the sample within reasonable time and resource constraints, convenience sampling was used. Further, previous research has found that over 50% of parents may be unaware of their adolescent’s self-harm behaviour and it was therefore expected that it would be difficult to recruit parents that are representative of all families experiencing this issue [37]. Parents were defined as anyone in a primary caregiver role for an adolescent. For ease of understanding, all participants are referred to as parents. Recruitment was conducted through targeted advertisements (including fliers, posters, and social media posts) to child and adolescent community mental health services, other community groups and social media parenting pages, where there was an expected reach of parents and carers of children who had engaged in self-harm behaviours. The researchers directly contacted the services and groups to advertise. Advertisements provided a link to an online survey where parents and carers were provided with information about the study and were able to self-select to be involved in the study. Recruitment occurred between March 2019 and May 2020. Seventy-seven participants entered the online survey using the link provided to them from advertisements. Inclusion criteria included being a parent of a child (12–18 years of age) who has engaged in self-harm behaviours. Of the 77 participants, 40 were excluded from the study (two participants chose not to provide consent, 37 participants consented but did not answer any questions, and one participant was excluded as their child was of adult age). Thirty-seven participants consented and completed the study, but ten of these participants only provided information on their adolescent and their response style, not demographic information. All participants were fluent in English.

### 2.2. Procedure

Once entering the survey, participants provided informed consent following approval of the local Institutional Review Board. To best understand the research questions, both closed- and open-ended questions were used to elicit quantitative and qualitative data to interpret trends and themes in participants’ experiences.

### 2.3. Materials

#### 2.3.1. Demographic and Clinical Questions

Parents were asked to provide demographic information in addition to information regarding their adolescent who self-harmed including age of onset of self-harm, method, number of methods, and frequency of self-harm. Due to reporting by parents, age, method, and frequency may not represent the experience of the child accurately and instead reflects parents’ perception of self-harm behaviour. Mental health diagnoses and mental health treatment of their adolescents were also provided by participants. Participants were asked to confirm if they had personally sought treatment for mental health.

#### 2.3.2. Mental Health Inventory 5 (MHI 5)

The MHI-5 is a brief screening measure for depression and anxiety [38]. It contains five questions measured on a scale from 1 (all of the time) to 6 (none of the time). Raw scores are summed and transformed to 0–100, with higher scores representing better mental health. The MHI-5 has been found to have good internal consistency with a Cronbach’s alpha between 74 [39] and 83 [38]. This measure has also been found to be a valid screening tool for mood and anxiety disorders [38,39]. While there is no formal cut-off for the MHI-5, several studies have used it to distinguish between ‘mentally well’ and ‘mentally ill’ participants. Cut-off points range between 52 and 76 [38,40,41,42]. One study investigating multiple cut-off points indicated that a cut-point of 60 could differentiate those with ill mental health from those with good mental health [43], and we adopted that here.

#### 2.3.3. World Health Organisation-Quality of Life (WHO-QOL)

Two questions from the WHO-QOL assessed self-reported quality of life and health satisfaction among respondents [44,45]. Item G1 asked “How would you rate your quality of life?” from 1 (very poor) to 5 (very good) and G4 asked “How satisfied are you with your health?” from 1 (very dissatisfied) to 5 (very satisfied). The WHO-QOL has good validity with an overall comparative fit index with quality of life of 0.97. Internal consistency has also been found to be good across the 6 domains measured in the WHOQOL with Cronbach’s alpha ranging from 0.71–0.86, and test–retest reliability was also good with correlations across domains ranging from 0.68–0.95 [44]. 

#### 2.3.4. World Health Organisation Disability Assessment Schedule 2.0 (WHO-DAS 2.0)

Two items from the WHO-DAS 2.0 were used to assess the impact of difficulties on participants’ lives [46]. Items H2 and H3 asked “in the past 14 days, how many days were you totally unable to carry out your usual activities or work because of any health condition?”, and “not counting the days that you were totally unable, for how many days did you cut back or reduce your usual activities or work?”. The WHO-DAS 2.0 has been found to have good construct validity and test–retest reliability ranged from acceptable to very good. This measure was found in our study to have good internal consistency, with a Cronbach’s alpha of 0.78.

#### 2.3.5. Relationship Stress Questionnaire-Parent Version (RSQ-P)

The RSQ-P is a self-report questionnaire developed to measure an individual’s response to stress [27]. Participants were asked to consider their responses to the stress of their child’s self-harm and answered 57 items on the frequency of responses on a scale from 1 (Not at all) to 4 (A lot). The RSQ-P has been found to be a reliable and valid measure of voluntary and involuntary stress responses [27]. Internal consistency across measures is acceptable with parent reports ranging from 49 to 78 and mean test–retest reliability coefficient = 65. Measures also had adequate convergent and divergent reliability in assessing stress responses [27]. Sample questions from the RSQ-P include “When dealing with stress as a parent I tell myself that everything will be all right” and “When dealing with the stressor, I feel sick to my stomach or get headaches”. The conceptual model of the RSQ includes voluntary coping styles and involuntary responses to stress. Within voluntary coping methods, there is both disengagement coping (e.g., avoidance, denial, wishful thinking) and engagement coping, which is further made up of primary control engagement (altering the problem, e.g., problem solving, emotion regulation) and secondary control engagement (adapting to the problem, e.g., positive thinking, cognitive restructuring, acceptance, distraction). Within involuntary response styles, there is involuntary engagement (e.g., physiological arousal, rumination) and involuntary disengagement (e.g., emotional numbing, cognitive interference, immobilisation). Scores for each type of coping are calculated as a proportion of the RSQ total score, to account for baseline differences. Means of all ratio scores were calculated to determine the average ratio for each coping style in this sample.

#### 2.3.6. Developed Survey Questions

Open-ended questions in the survey allowed a chance for parents to reflect on the impact of their child’s self-harm. Questions included: (1) Can you describe what effects, if any, your child’s self-harm has had on you?; (2) Can you describe what effects, if any, your child’s self-harm has had on your family?; (3) Can you describe what effects, if any, your child’s self-harm has had on your involvement in the community and with friends? Parents were asked to respond in four or more sentences to the three questions.

### 2.4. Data Analysis

Quantitative and qualitative data were analysed and interpreted separately to address the aims [35,36]. Quantitative data was analysed using SPSS Version 25. Pearson zero-order correlations were conducted to understand the relationship between perceived severity and frequency of self-harm and quality of life, health satisfaction and mental health of parents. Pearson zero-order correlations, and independent *t*-tests with Bonferroni correction (adjusted *p* value = 0.01) were conducted to understand the relationship of parent response style and quality of life, health satisfaction, days out of role, and mental health. All assumptions for quantitative analyses were met.

Qualitative survey responses were imported into NVivo 12 for analysis. Thematic analysis was used in this study to identify and understand patterns of meaning in the dataset [47]. Using an inductive approach to analysis, the authors familiarised themselves with the data by re-reading the responses multiple times. One author then generated initial codes which were then examined in cross coding sessions where the authors documented the emergent codes in a coding frame. Candidate themes were mapped through a recursive process and final themes were defined after refinement [48]. Quotes were then selected for the manuscript that are exemplar of the data belonging to a particular theme. All parents provided qualitative data and all responses were included in the qualitative analysis. The average length of the parents’ individual question responses was 58 words (*SD* = 12.8).

## 3. Results

### 3.1. Quantitative Responses

Thirty-seven participants consented and completed the survey, but 10 did not provide demographic information. The mean age of parents was 45.70 (*SD* = 6.18). Most participants were born in Australia (77.8%), one participant identified as Aboriginal, and there was diversity in annual income across the participants. Table 1 provides demographic information. 

All participants answered questions regarding their children. Missing data was missing completely at random and imputed using expectation maximisation. Most adolescents who had self-harmed were female (75.7%). The average current age of participants’ children was 16.89 (*SD* = 3.91), and perceived age of onset of self-harm, as reported by parents, was estimated as 12.73 years (*SD* = 2.28, range = 5–18 years). Although some participants’ children began to self-harm during childhood, all self-harmed during adolescence, and questions were specific to adolescent self-harm as reported by parents. Parents indicated that 88.9% of adolescents had experienced a mental health problem and 86.5% had received treatment for self-harm. There was variation in the method, number of methods, frequency of self-harm, and mental health diagnoses (Table 1).

#### 3.1.1. Mental Health and Psychosocial Functioning of Parents

The average score on the MHI-5 for the sample was 50.96 (*SD* = 20.50). Using the cut-off point of 60 on the MHI-5, 66.7% of the sample were categorised as likely to have mental ill-health. Better mental health, as measured by the MHI-5, was positively correlated with quality of life (*r* = 0.705, *p* = < 0.001), and health satisfaction (*r* = 0.530, *p* = 0.001).

Many (70.4%) of the participants had sought treatment for their own mental health. Of those that had sought help, 84.2% had engaged in therapy and 84.2% had taken medication. Of the eight participants who had not sought mental health treatment, five scored in the mentally ill category on the MHI-5.

Participant quality of life varied, with 37% of participants reporting that their quality of life was neither poor nor good (Table 1). With regards to health satisfaction, a third (33.3%) of participants were somewhat dissatisfied with their health, and 25.9% were somewhat satisfied. On average, participants had 1.81 days where they were totally unable to carry out their normal work or activities in the past two weeks (*SD* = 3.21), and 4.33 days of reduced capacity to function in the past two weeks (*SD* = 5.27).

#### 3.1.2. Relationship between Perceived Severity and Frequency of Adolescent Self-Harm and Parent Mental Health and Psychosocial Functioning

On the RSQ, the biggest stressor endorsed by parents was ‘arguing with your child or children’ (*M* = 3.25, *SD* = 0.83). Mean ratio scores for RSQ domains and correlations with mental health and quality of life are presented in Table 2.

In this sample, the most common participant response to stress was involuntary engagement coping (*M* = 0.28, *SD* = 0.05). For the majority of participants (n = 29) this was their most common response, characterised by rumination, intrusive thoughts, physiological arousal, emotional arousal and involuntary action. Involuntary engagement coping was negatively correlated with mental health (*r* = −0.647, *p* = < 0.001), quality of life (*r* = −0.388, *p* = 0.046) and health satisfaction (*r* = −0.422, *p* = 0.028). The second most common response to stress was secondary control coping (*M* = 0.23, *SD* = 0.05) which includes positive thinking, cognitive restructuring and acceptance. Secondary control coping was positively correlated with mental health (*r* = 0.792, *p* = < 0.001), quality of life (*r* = 0.682, *p* = < 0.001) and health satisfaction (*r* = 0.504, *p* = 0.007). While participants may have used one type of coping more frequently than others, all coping styles were correlated with each other, which indicates that most individuals use several coping strategies (Connor-Smith et al., 2000). Primary and secondary control coping were positively correlated, and both were negatively correlated with voluntary disengagement coping, involuntary engagement and involuntary disengagement coping styles.

Responses of participants who primarily used involuntary engagement coping were compared to those who used secondary control coping using independent *t*-tests with Bonferroni correction. The participant group who primarily relied upon involuntary engagement coping had significantly lower quality of life, *t* = 3.80, *p* = 0.001, CI (0.68, 2.31), Cohen’s d = 2.13. Based on MHI-5 scores, the parents using secondary control coping (*M* = 80.80, *SD* = 3.35) had significantly better mental health than those using involuntary engagement coping (*M* = 43.81, *SD* = 16.37), *t* = 4.95, *p* = < 0.001, CI (21.58, 52.40), Cohen’s d = 3.16. As the Bonferroni correction was applied, there was no significant difference in health satisfaction, *t* = 2.44, *p* = 0.022, CI (0.22, 2.55), days out of role, *t* = −0.759, *p* = 0.455, CI (−4.64, 2.14), and days of reduced role function, i = −2.47, *p* = 0.023, CI (−6.90, −0.58).

### 3.2. Qualitative Responses

Thematic analysis of the open-ended survey questions resulted in five themes being identified and three sub themes. Illustrative quotes are presented that are typical of the data that belongs to a particular theme. Table 3 provides an overview of the themes and further details are provided in-text.

#### 3.2.1. Sustained Feelings of Parental Distress and Fear

Participants reported ongoing aversive feelings in relation to their child’s self-harm. Parents indicated their lives were characterised by feelings of worry and anxiety, stress, and strain. Often, this was coupled with feelings of sadness, frustration, anger, and confusion around why their adolescent would engage in self-harm.

The most common content of distress and fear reported by participants was worry about the safety of their adolescent, and fear that their child may seriously injure themselves unintentionally during an episode of self-harm. Participant #22 remarked “*I live with constant stress and worry about when it could happen again*” while participant #60 noted “*it is incredibly painful as a parent to know that your child is in such emotional pain that they would resort to cutting their own skin*”. Some parents further expressed worry about the young person’s future—fearing they will regret their current actions and will face discrimination, “*As parents we fear for his future. How much worse will things get after puberty? Will he cope going to high school? He is a very bright boy but we are concerned that he won’t reach his potential*” (Participant #68).

For the participants in this study, the ongoing distress and fear contributed to negative evaluations regarding their own effectiveness as a parent and hope for their adolescents. Many participants discussed intense feelings of shame, self-blame and the belief that they have failed as a parent. Some parents noted that feelings of failure came from feeling responsible for their adolescent’s self-harm, while others indicated that they felt they had let their adolescent down by not providing enough help. Participant #14 reported “*It has made me very upset to feel she is hurting so much she feels the need to harm herself. I feel like I have let her down, haven’t helped her enough and made her the way she is*” and participant #33 commented “*My heart is weak, not physically, but emotionally. I feel like I have failed as a parent for not being able to keep my child safe. Even though my intellect knows that I have done everything that I possibly could to care for her*”. These feelings may have further exacerbated parents’ reported experiences of isolation, helplessness, and hopelessness.

#### 3.2.2. Behavioural Reactions to Ongoing Distress and Fear

Parents described a sense of hypervigilance towards their adolescent who was self-harming. Participants reported they were always highly aware of the needs of their young person and were constantly monitoring their mood and level of risk. For example, participant #22 noted “*I have to monitor and supervise my son everyday and be mindful of his needs, triggers, stressors and keep risks to a minimum*”. Parents also described more active forms of supervision, where they were afraid to leave their adolescent alone. For some parents, this constant need to monitor and be aware of subtle cues from their adolescent led to anticipatory dread—always bracing themselves for another episode of self-harm or other injurious behaviour. Participant #40 reported “*I’m very nervous now when I haven’t heard from her for a few hours. My anxiety is always high regarding her*” while another parent said, “*every time I walk into the house I brace myself, not knowing if she has harmed herself while I have been out*” (Participant #33). Participants noted that the necessity of constant monitoring and supervision was exhausting and isolating. This then had subsequent impacts on the parent, particularly on their ability to function effectively in various realms. Parents withdrew from social circles and events and reduced or stopped work in order to maintain a capacity to monitor and supervise their adolescent.

#### 3.2.3. Impact of Adolescent Self-Harm on Parent Mental and Physical Health

Several participants indicated that adolescent self-harm had impacted their own mental and physical health. Parents noted diagnoses of mood disorders arising from ongoing worry and stress including “*I myself now have anxiety, depression and trauma from dealing with her self-harm*” (Participant #38). Some participants reported an increase in physiological arousal including difficulties sleeping and nausea, with participant #31 stating “*I can’t eat properly as feel so sick and my stomach so painful, I can’t sleep, I have horrendous anxiety all the time*”, and two participants linked difficulties with their physical health to stress regarding their child’s self-harm.

#### 3.2.4. Change in Family Dynamics

Subtheme: Change in dynamics of immediate family relationships. Participants reported a significant change in the interactions among immediate family members when an adolescent was engaging in self-harm. Notably, several participants indicated that family members were hypersensitive to the adolescent, modified interactions so they reduced the likelihood that the adolescent may become distressed and self-harm, and therefore changes in the nature of relationships. Participant #70 commented “*We are all very aware that she is not in a good way and we are ‘treading on eggshells’*”. For some participants, this resulted in a reduced ability to set boundaries and deliver consequences when necessary, as participant #18 reflected, “*I’ve become a push over. I find it hard to say no*”.

The impact of adolescent self-harm on siblings was varied. Some parents noted that siblings felt excluded and found the differing expectations on them versus the adolescent who was self-harming were unfair. Several parents indicated that siblings felt anxious and stressed about their sibling who was self-harming. Participant #68 commented “*One child has had trouble sleeping so I asked her to write down what she was worried about and give it a rank out of 10. She gave her brother’s situation a 10/10. As a parent your heart breaks all over again because of the impacts on other members of the family. They have asked what is wrong with him*”. Often, this seemed most pronounced in younger siblings. Lastly, some participants reported that siblings disengaged or withdrew, avoiding the topic and at times family interactions, “*our son who has witnessed a lot of our daughter’s self-harm, he is very withdrawn and has never spoken to us about what was happening, we have tried to talk to him about it but he will not engage*” (Participant #51). One parent acknowledged withdrawal by the sibling was an effort to protect themselves—“*anxiety with sibling, but also ignoring to protect self to some degree*” (Participant #19).

Subtheme: change in spouse relationship. Seven participants reported adolescent self-harm had contributed to spouse relationship strain. Relationship strain and changes to spouse relationships included withdrawing from spouse, increased conflict with spouse, tension due to different parenting approaches and in some cases relationship breakdowns. One participant indicated that “*always being aware of her behaviour has put a strain on the family and contributed to my marriage breakdown*” (Participant #10). Other participants indicated that their spouse found adolescent self-harm difficult to manage, becoming withdrawn or angry.

Subtheme: change in extended family relationships. The impact of adolescent self-harm on the extended family appeared dependent on the way individuals responded to the adolescent self-harm. Some participants noted their child’s grandparents and extended family experienced similar feelings to the parents—worry, stress, sadness, helplessness—and “*they do not know what they can do to help us. They are willing to help where they can, her self-harm has distressed them*” (Participant #33). Other participants reported that adolescent self-harm contributed to conflict and tension among extended family, often leading to feelings of distance or estrangement from previously close family members. Participants noted that the main cause of distance was extended family not understanding adolescent self-harm; participant #3 said “*(Adolescent’s) mental health has put a lot of stress on the family, we don’t see them that much anymore. We don’t want to cause them unnecessary stress and they don’t want to understand the mental health condition*”.

#### 3.2.5. Functional and Social Impacts of Adolescent Self-Harm on Parents and Families

There were several functional impacts reported of adolescent self-harm on parents and the wider family. Several participants reduced their work hours or stopped work completely to provide care for the adolescent, with an associated financial impact on the whole family. One participant reflected that “*I thought I would be working full-time by now. But I have been her ‘Carer’, on a low pension. So I have no superannuation. So even when she moves out, eventually, I will need to do a quick catch-up*” (Participant #33). Some parents relocated in order to be closer to appropriate services for the young person. Participants also refrained from making significant changes in their lives or put their goals on hold in order to care for their young person. For example, participants reported they “*push[ed] back my studies*” (Participant #34), did not pursue intimate relationships as “*I’m not going to start another one while she is still at home with me*” (Participant #25), and “*put (trying to conceive) on hold for ten years to deal with her mental health issues. Now it looks like we left it too late*” (Participant #40).

Many participants discussed adolescent self-harm having an impact on their capacity to engage socially and in the community. Several participants noted they had drifted from their own support networks of friends either because they were unable to leave their child alone or because their friends had difficulty understanding the behaviour of the child who self-harms. Participant #3 reported “*I have alienated myself from my friends as I’m always worried about my daughter’s wellbeing, so I feel I’m not that fun to be around, plus I feel like I need to be available for my daughter if she ever needs that extra support, so I try not to plan outings that don’t involve her*.” More broadly, participants indicated the most significant limitation to their social function was the fear that they could not leave their adolescent alone unattended for any length of time, in case they engaged in injurious behaviours. Participants noted they did not even try to leave the house anymore, or if they did, they stayed close to home in case their adolescent became distressed, and they needed to leave. Participant #33 reported “*I have difficulty committing to social events because I do not feel secure leaving her unattended*” while participant #15 noted “*I have withdrawn from socialising as much as I would like to as I am the sole carer for my daughter and when I am not attentive she can do unhealthy things. I don’t participate in many community events because I don’t want to be absent from home. I don’t want to force my daughter to come out with me because she finds it hard to socialise and this may increase her distress and therefore her ‘need’ to self-harm*”.

Several participants noted they did not receive any support from friends or family. There were various reasons given for this, including previous poor experiences with disclosing adolescent self-harm to family or friends—“*(I) don’t ask for help. I don’t get emotional support from my family*” (Participant #22), thinking that no one is able to provide help and so there is no use in discussing their adolescent, or feeling embarrassed or ashamed as a parent*—*“*I was far too ashamed to tell anyone*” (Participant #60) and not wanting to betray their child’s trust and privacy.

## 4. Discussion

This study aimed to provide an in-depth understanding of parent perceptions of the impact of adolescent self-harm, and how adolescent self-harm impacts on parent and wider-family wellbeing and community involvement, from the perspective of parents. We also aimed to understand the typical stress response style of parents towards their adolescent who self-harms, and the relationships between stress response style and the impact of adolescent self-harm on parents in terms of distress and functional impairment. We hypothesised that participants would have experienced psychological distress and dysfunction. We further hypothesised that parents who typically employ less adaptive stress response styles will have experienced greater distress and dysfunction compared to parents who are able to respond in a more helpful manner. In addition, we used qualitative responses to understand parents’ perspectives of the impact of adolescent self-harm on themselves, their family, and their social and community engagement.

In line with our first hypothesis, the parents in this study experienced distress and functional impairment with the number of self-harm methods perceived by parents related to greater distress and poorer functioning. Two thirds of parents were categorised as likely to have mental ill-health according to the MHI-5 and 70.4% had sought treatment for their own mental health. Interestingly, not all participants who scored in the mentally ill category on the MHI-5 had sought treatment. This could reflect the numerous barriers to treatment that parents face when experiencing difficulties with mental health or caregiver burden, including perceived need for intervention, desire to manage their mental health alone, shortage of time, prioritising other family members, financial strain and fear that they might be subject to stigma for seeking help [49,50]. Further, given the sample consisted of parents who were aware of their adolescent’s self-harm, and there are often barriers to disclosure from parents [37], it may be that the barriers to seeking mental health treatment are even more significant than those reflected in this sample. This may be important to address, as parental mental health difficulties can impair the capacity for effective parenting. Relatedly, parent psychopathology has been associated with several aversive outcomes for children including internalising, externalising and attachment difficulties, and the development of BPD [16,17,18]. It is therefore important to address barriers that parents may face in seeking their own help to reduce the likelihood of ineffective parenting and transmission of mental health difficulties to children.

Results indicated that participants mostly relied on involuntary engagement coping when responding to stress, followed by secondary control coping. Involuntary engagement coping is characterised by rumination, intrusive thoughts, physiological arousal, emotional arousal and involuntary action (e.g., ‘I can’t control what I say or do’), while secondary control coping is characterised by adapting to the problem by using skills such as acceptance, positive thinking and cognitive restructuring [27]. Our second hypothesis was supported; parents in this sample who used involuntary engagement coping the most had significantly poorer mental health and quality of life—but not poorer functional capacity—compared to those who used secondary control coping. This suggests that the stress response style of parents in this sample may be related to their psychological health. Therefore, it may be that parents who are better able to use realistic thinking, perspective taking, defusion from negative thoughts and acceptance, are perhaps better able to tolerate ongoing stressors like adolescent self-harm, and therefore reduce the impact on their own health.

Further investigation is needed to understand the direct relationship between adolescent self-harm and parent response style and several factors that we were unable to account for could explain some of the variance in results. However, these findings suggest that one way to support an adolescent who is self-harming may be to provide assistance or support to the parents. When considering the distress and impairment evident in our parent sample, and the qualitative responses which detailed the significant impact adolescent self-harm has on parents, our results suggest that parents perceive the impact of adolescent self-harm on themselves to be significant. These results are in-line with family systems theory which emphasises the dynamic relationship of stressors within the family and the reciprocal impact they can have on family members [13,14]. Parents therefore require support to maintain their own health, and some parents may face barriers in accessing this support. Future studies could aim to recruit a control group in order to identify more specifically the relationship of adolescent self-harm to parental wellbeing, compared with other child mental ill-health difficulties or with a healthy group of adolescents.

Our results suggest that parent response style may impact on parent wellbeing, and response styles to stress may be amenable to therapeutic intervention, which could include reducing the reliance on involuntary disengagement coping and improving the ability to use primary or secondary control coping [26,29]. This may be important for psychologists and other mental health professionals to consider when providing psychological treatment for an adolescent. Encouraging the importance of parents seeking their own mental health treatment may help to increase the parent’s capacity to appropriately respond to and support their adolescent. Cognitive behaviourally based interventions typically focus on cognitive restructuring and behaviour modification in order to target rumination, negative attribution styles and aversive physical and emotional states [51,52]. They often also incorporate mindfulness-based strategies, which were highlighted by Whitlock et al. [22] as inversely related to caregiver strain. Developing an individual or group-based cognitive behavioural intervention focusing on shifting from unhelpful thoughts, feelings, and behaviours towards more adaptive ways of understanding and living in their environment may serve as a buffer to protect parent’s own health. This may then have a follow-on effect, where parents who are mentally and functionally well are better able to meet the needs of their adolescent who self-harms. This is in-line with family systems theory which emphasises the need to provide psychological support to the family in order to address the interrelated processes occurring within the family environment [13].

The results of qualitative analysis highlight the pervasive impact of adolescent self-harm on parents’ lives. Parents experienced ongoing distress and fear, coupled with hypervigilance, anticipatory dread, and a decrease in their own mental health. These results reflect findings from previous research [19,21,23,24,25,34], reiterating the feelings of distress and fear in parents whose adolescent self-harms and continuing to highlight the unmet needs of this group of parents. The qualitative analysis also contributed to a further understanding of the impact of adolescent self-harm on the wider family and social engagement. Adolescent self-harm significantly altered family dynamics, with reactions of anxiety and stress or withdrawal and disengagement common. These understandable reactions to confronting issues may be perceived by adolescents with a hypersensitive temperament or negative attentional bias as rejection or criticism [53], which may in turn perpetuate adolescent self-harm as a way to reduce negative affect [4]. It is possible that acknowledging the impact on the wider family, with a family-system approach to treatment may help to identify ways in which family members can assist, while continuing to care for themselves and maintaining appropriate boundaries, and can support siblings. Finally, adolescent self-harm was found to negatively impact on social and community engagement and functioning for families. Providing extra functional support, such as financial support or access to support groups to encourage community engagement to parents of adolescents who self-harm, could help address some of these impacts.

This study has several limitations. Firstly, due to time and resource limitations our study employed convenience sampling to collect data from a significant sample size. However, this may limit the generalisability of the results and may not accurately reflect the entire population of parents of a self-harming adolescent. While this study provides some evidence of a relationship between parenting and self-harm in adolescents, future studies could aim to recruit community samples to further explore this relationship.

Moreover, data detailing adolescent self-harm (including age of onset, method, and frequency of self-harm) were reported by parents and may therefore not accurately represent actual age of onset, method, and frequency, depending on the information known to the parent. Previous research has found discrepancies between adolescent and parent reporting of self-harm behaviour with more than 50% of parents being unaware of their child’s self-harm behaviour, and 50% of adolescents indicating they did not disclose self-harm behaviour to their parents [37]. Therefore, parents may not be aware of all aspects of adolescent self-harm behaviour. Similarly, perceptions of the impact to parent and family wellbeing were provided through online parent self-reporting and may display personal biases. Adolescent self-reported, or clinician-reported data may provide further representation and these results should be interpreted tentatively in our study. However, the aims of this study specifically investigated perceptions of parents and the impact on wellbeing and stress-response, therefore the data collected is still highly beneficial to understanding the drivers of stress and impacts on wellbeing for parents. Further, the use of both quantitative and qualitative data was a strength of this study as it provides different approaches and a deeper understanding of how adolescent self-harm and parents’ responses to this affects parents’ wellbeing and functioning, as well as the wider family.

Further, our study included no direct measure of parents’ perception of severity; instead parents indicated the number of methods their child used. Similarly, our study did not investigate lifetime frequency of self-harm or whether parents perceived their adolescent to also have suicidal ideation or behaviour. While we found there to be no relationship between perceived frequency in the last 12 months and adult wellbeing and mental health, these other factors could be associated with response style and mental health and wellbeing of parents, and future studies could aim to investigate this further. Future studies should also consider using a comparison group of parents whose child does not have a self-harm history.

When considering the interactional nature of parent–child relationships and previous research, it seems likely that parenting style influences the development and maintenance and potentially the cessation of adolescent self-harm, and in turn this self-harm then impacts on the response style of parents, which then may further influence the adolescent’s self-harm behaviours. This supports a family system theory perspective which emphasises the dynamic relationship between family members and the interdependence of parents and their children and highlights the importance of acknowledging and targeting the ways in which behaviour and emotions and others’ responses to behaviour and emotions are reciprocal [13,14]. As this was a cross-sectional study focused on parent perspectives only, it was not possible to gauge the impact of stress response style on adolescent behaviours, including self-harm. This may be an important area for future research to understand the within-family interactions that may perpetuate—or alternatively, alleviate—difficulties with self-harm.

The sample size was modest and future work with diverse and larger populations may add to the results presented, including sampling more male parents. Further, mental health of parents was measured using a screening instrument for distress, and as such there may be disorder-specific relationships that were not able to be identified in this sample. As our research showed that mental health was associated with response style of parents, future research could explicitly investigate the impact of specific mental health disorders on response style and adolescent self-harm.

Future studies could further investigate the role of stress response style in parents of adolescents who self-harm to determine how parent response style could both be an effect of adolescent self-harm and potentially foster adolescent self-harm. It may be helpful to evaluate the impact of response style over time and the direct impact of parent response style on adolescent self-harm and mental health. Following on from this, interventions focusing on supporting the parents of adolescents who self-harm may be useful in helping them alter response styles, thus possibly reducing parenting burden and perhaps influencing the adolescent’s self-harm behaviour.

## 5. Conclusions

This study identified that parents of adolescents who engage in self-harm experience significant distress and functional impairment. Parents who responded to stress with maladaptive coping had significantly poorer mental health and quality of life compared to parents who were able to respond to stress with acceptance, positive thinking, and cognitive restructuring. Qualitative analysis confirmed the impact of adolescent self-harm on parents and highlighted the impact on the dynamics of the wider family including a reduced capacity to meaningfully engage in social activities. When considering adolescent self-harm, support for parents and the wider family should be considered by health professionals.

## Figures and Tables

**Table 1:** Demographic and clinical information.

		N ^1^ (%)
Relationship to child	Mother	33 (89.2)
	Father	2 (5.4)
	Stepmother	1 (2.7)
	Foster carer	1 (2.7)
Relationship status	Married	9 (33.3)
	De-facto	6 (22.2)
	Single	4 (14.8)
	Divorced	4 (14.8)
	Separated	4 (14.8)
Current employment status	Work or study full-time	16 (59.3)
	Work or study part-time	6 (22.2)
	Not currently working or studying	5 (18.5)
Education	Did not complete high school	4 (14.8)
	Completed high school	1 (3.7)
	Trade or vocational school	9 (33.3)
	University degree	13 (48.1)
Number of children in family	1	5 (19.2)
	2	10 (38.5)
	3	10 (38.5)
	>4	1 (3.8)
Annual household income (AUD)	<$20,000	5 (18.5)
	$20,000–$40,000	3 (11.1)
	$40,000−$60,000	2 (7.4)
	$60,000−$80,000	7 (25.9)
	$80,000−$100,000	5 (18.5)
	$100,000−$120,000	1 (3.7)
	$120,000−$140,000	4 (14.8)
	$140,000−$160,000	1 (3.7)
	>$160,000	2 (7.4)
Parent quality of life	Poor	7 (25.9)
	Neither poor nor good	10 (37.0)
	Good	7 (25.9)
	Very good	3 (11.1)
Parent health satisfaction	Very dissatisfied	3 (11.1)
	Somewhat dissatisfied	9 (33.3)
	Neither satisfied nor dissatisfied	5 (18.5)
	Somewhat satisfied	7 (25.9)
	Very satisfied	3 (11.1)
Perceived adolescent method of self-harm ^2,3^	Cutting	33 (89.2)
	Scratching	12 (32.4)
	Hitting	11 (29.7)
	Burning	6 (16.2)
	Other	13 (35.1)
Perceived number of adolescent methods of self-harm ^2^	1	11 (29.7%)
	2	17 (45.9%)
	3	5 (13.5%)
	4	1 (2.7%)
	5	2 (5.4%)
	6	1 (2.7%)
Perceived adolescent frequency of self-harm in last year ^2^	Daily	3 (7.9)
	Weekly	6 (16.2)
	Monthly	8 (21.6)
	Quarterly	7 (18.9)
	Once or twice	4 (10.8)
	Nil	9 (24.3)
Adolescent diagnosis of mental health disorder	Depressive Disorder	22 (59.5)
	Anxiety Disorder	19 (51.4)
	Borderline Personality Disorder ^4^	15 (40.5)
	No diagnosis	6 (16.2)
	Post-Traumatic Stress Disorder	6 (16.2)
	Attention Deficit Hyperactivity Disorder	4 (10.8)
	Complex Post Traumatic Stress Disorder	3 (8.1)
	Autism Spectrum Disorder	3 (8.1)
	Obsessive Compulsive Disorder	3 (8.1)
	Eating Disorder	3 (8.1)
	Psychosis	2 (5.4)
	Bipolar Disorder	2 (5.4)
	Oppositional Defiance Disorder	2 (5.4)
	Gender Dysphoria	1 (2.7)

^1^ Total N differs between questions depending on how many participants answered questions within the survey. ^2^ Method and frequency of adolescent self-harm was provided by parents and may not reflect actual method and frequency. ^3^ Several participants noted that their children engaged in more than one form of self-harm. ^4^ Includes BPD traits.

**Table 2:** Response style by parents measured using the Relationship Stress Questionnaire and its relationship to mental health and quality of life.

Response Style (Mean Ratio)	*M* (*SD*)	Mental Health	Quality of Life
Primary control coping	0.18 (0.04)	0.51	0.30
Secondary control coping	0.23 (0.05)	0.82 *	0.72 *
Disengagement coping	0.14 (0.03)	−0.38	−0.21
Involuntary engagement coping	0.28 (0.05)	−0.66 *	−0.42
Involuntary disengagement coping	0.17 (0.04)	−0.520*	−0.60 *

*Note.* Mental health = Mental Health Inventory-5 (MHI-5) score. Quality of life = World Health Organisation Quality of Life (WHO-QOL) score. * *p* < 0.005.

**Table 3:** The impact of adolescent self-harm on parents.

Themes and Subthemes	Overview of Themes
Sustained feelings of parental distress and fear	Reporting of aversive feelings in relation to self-harm of adolescent Worry about adolescent’s safety Negative evaluation of own effectiveness as parent
2. Behavioural reactions to ongoing distress and fear	Hypervigilance towards adolescent High monitoring and supervision of adolescent
3. Impact of adolescent self-harm on parent mental and physical health	Diagnoses of mood disorders Increase in physiological arousal
4. Change in family dynamics Subtheme: Change in dynamics of immediate family members Subtheme: Change in spousal relationship Subtheme: Change in extended family relationships	Hypersensitivity and adjusted behaviour towards adolescent Impact on the nature of relationships among immediate family members Adverse impact on siblings of adolescent Spouse relationship strain, including relationship breakdown Feelings of stress and sadness experienced by extended family members Conflict and tension among extended family members
5. Functional and social impacts of adolescent self-harm on parents and families	Capacity to work, and finances, impacted Relocation Adolescent care prioritised over personal objectives Capacity to engage socially, and in the community, impacted Limited support from friends and family

## Data Availability

The datasets generated and/or analysed during the current study are not publicly available but are available from the corresponding author on reasonable request.
